# Spectral Cruncher: A Visualization Tool Integrating Manual Curation, Ion-Intensity Prediction, and De Novo Tag Generation

**DOI:** 10.1021/jasms.5c00301

**Published:** 2025-12-29

**Authors:** Aline A. M. Martins, Blake L. Tsu, Hulyana Brum, Lucas Sales, Marlon Dias Mariano dos Santos, Juliana de Saldanha da Gama Fischer, Stephanie Almeida, Luisa Bulcao Vieira Coelho, Natalia Moreira, Alysson R. Muotri, Paulo Costa Carvalho

**Affiliations:** † Integrated Space Stem Cell Orbital Research Center, 8784 University of California San Diego, La Jolla, San Diego, California ZIP: 92037, United States; ‡ Laboratory for Structural and Computational Proteomics, Carlos Chagas Institute–Fiocruz Paraná, Curitiba; ZIP: 81350-010, Brazil; § Analytical Biochemistry and Proteomics Unit, Instituto de Investigaciones Biológicas Clemente Estable/Institut Pasteur, Montevideo; ZIP: 11400, Uruguay

**Keywords:** Spectral, interactive, PatternLab, designed, tag, intensity, SpecFormer, instrument-specific, data, sets, providing, Q-Exactive+, bulk, Astral, single-cell, bulk, tools, available

## Abstract

Here, we introduce Spectral Cruncher, an interactive extension to the PatternLab for Proteomics platform, designed to bridge the gap between manual curation and state-of-the-art computational analysis of proteomic tandem mass spectra. Spectral Cruncher integrates de novo sequence tag extraction, automated spectral annotation, targeted tag search, and a customized transformer-based fragment-ion intensity predictor (SpecFormer) within a unified graphical environment, designed for interactive and instrument-specific visualization. Central to this workflow is SpecFormer, a compact transformer architecture trained on multiple data sets, providing independent ion intensity models for Q-Exactive + bulk, Astral bulk, and Astral single-cell proteomics data, enabling accurate and instrument-specific intensity prediction even under conditions of sparse fragmentation and low signal-to-noise ratios. Evaluation of SpecFormer demonstrates high predictive performance, with average cosine similarities of approximately 0.98 for bulk Q-Exactive + data sets, 0.91 for bulk Astral, and 0.87 for Astral single-cell data. These tools enable researchers to interrogate ambiguous spectra, validate peptide identifications, and develop intuition for algorithmic limitations. The tools are freely available within PatternLab 5.1, lowering technical barriers and promoting broader adoption of interactive, expert-driven workflows as well as providing a learning environment. A video of our tool in action is available at https://youtu.be/tc2sPiqJkLA.

## Graphical abstract

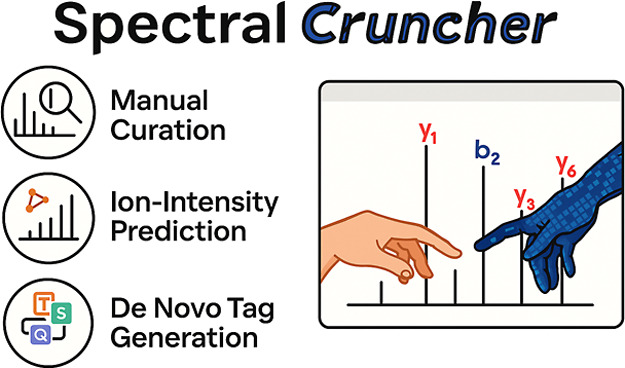

## Introduction

Before algorithms ruled the proteomics landscape, researchers developed an almost musical ear for spectral patterns, distinguishing real peaks from noise and identifying amino acid sequence tags through careful manual examination. Ask veterans in proteomics about their training, and many will fondly recall marathon sessions of manual spectral validation, a rite of passage that built deep intuition but has quietly faded from modern workflows. This evolution, while bringing powerful computational advances, has inadvertently created a critical gap: the art of manual spectral examination that once defined the expert proteomicists. While modern proteomic scientists benefit from sophisticated algorithms, the lack of visual and interactive tools for examining native spectra increasingly shadows opportunities to develop this fundamental skill and art. Modern platforms treat peptide-spectrum matching (PSM) as an opaque process, providing minimal tools for users to visually interrogate spectra, validate identifications, or investigate fragmentation patterns. This opacity not only hinders the manual curation of ambiguous spectra, particularly those involving novel PTMs, mutations, or atypical ion series that require expert scrutiny. Several specialized spectral viewers exist, including MS-Viewer for annotated database search results,^1^ interactive peptide spectral annotator (IPSA) for tandem mass spectrum annotation,^2^ and Spectroscape for real-time spectral archive queries.^3^ However, these tools rarely integrate into complete proteomics workflows or provide exploratory capabilities like visual de novo sequence tag generation and scoring. A more recent development, MZCal (2), provides a web-based and mobile-friendly interface for theoretical peptide calculations and spectral prediction using MS^2^PIP. While useful for visualizing fragment ions, MZCal functions primarily as a viewer and calculator and does not support the direct opening of raw files, or the generation of sequence tags.

In a similar vein, the proteomics field has seen a surge in artificial intelligence (AI) models for predicting fragment ion intensities, which unarguably has shown to improve PSM confidence in both data-dependent acquisition (DDA) and data-independent acquisition (DIA) pipelines. Notable examples include Prosit, a deep neural network for proteome-wide tandem mass spectrum prediction,^4^ and MS^2^PIP, which employs machine learning to forecast peptide fragmentation patterns across various instruments and proteases.^5^ Recent tools like DIA-BERT further leverage transformer-based AI for end-to-end DIA analysis^6^ and iDIA-QC uses AI for quality control in DIA workflows.^7^ However, these models are often instrument-specific, as fragmentation patterns vary between analyzers (e.g., linear ion traps, Orbitraps, etc.), and their integration into interactive environments remains underdeveloped. Systematic assessments highlight that while these predictors perform well, challenges persist in generalizing across data sets, particularly for low-abundance ions;^8^ in fact, in a recent study, the authors report that “in single-cell samples, low-abundance peptides derived from small protein contents can only provide limited fragment ions, which may distort the MS2 spectra. The different characteristics and relatively lower signal-to-noise ratio in SCP spectra may affect the software’s judgment in the spectrum matching process”.^9^

The evolution of AI in computational proteomics has progressively addressed these limitations by moving from early, hand-engineered feature models to architectures that learn richer fragmentation patterns and generalize better. Initial spectrum-intensity predictors relied on carefully crafted physicochemical descriptors paired with classical machine-learning algorithms. For instance, the 2013 release of MS^2^PIP used random-forest regression on amino-acid property vectors and achieved a median Pearson correlation of ≈0.75 on external CID, charge-2+ spectra. A 2019 rebuild adopting gradient-boosting (XGBoost) and larger training sets raised the median correlation to 0.90–0.95 for most fragmentation-specific models.^5^ In what followed, recurrent neural networks marked a pivotal transition: the pDeep series applied bidirectional long short-term memory networks (Bi-LSTMs) to also surpass 0.90 correlation across HCD, ETD, and EThcD spectra, with subsequent iterations introducing transfer learning for PTMs and few-shot fine-tuning for cross-instrument accuracy.^10^ Prosit advanced this further by coupling sequence-to-sequence LSTMs with massive data sets, adding retention time prediction with near-perfect rank correlation. The shift to transformer architectures, as in the Prosit Transformer and AlphaPeptDeep, a compact four-layer model with modular training, has proven superior for longer peptides and multitask learning (e.g., concurrent forecasting of retention time and ion-mobility collision cross-section), achieving correlations ≥0.90 for 97% of PSMs while handling over 21 PTMs.^11^

Despite these innovations, important gaps remain. Performance can degrade by 20–40% when collision-energy settings or instrument types differ between training and inference data sets, highlighting difficulties in cross-platform generalization. Single-cell proteomics exacerbates these hurdles, where low protein copy numbers (spanning up to 7 orders of magnitude dynamic range) reduce PSM confidence and ion detection.^9,12^ Additional complications include incomplete cysteine carbamidomethylation and diminished precursor ion abundance, necessitating tailored models to mitigate false discoveries. Missing-value rates can climb to 60–80%, eroding assumptions in most loss functions that expect dense measurement matrices. From a software perspective, most tools are simply short Python scripts demanding complex environments with versioning control pinned to narrow ranges of CUDA, cuDNN, that simply serve as wrappers to deep-learning frameworks generated in other languages. Maintaining these stacks requires administrative privileges, meticulous dependency pinning, and frequent patching, tasks that divert researchers from experimental design toward system maintenance.

PatternLab for Proteomics is a comprehensive computational platform for mass spectrometry-based protein identification and quantification, first introduced as one of the pioneering desktop tools for shotgun proteomics analysis.^13–15^ Since its inception, PatternLab has undergone continuous updates, evolving into an integrated workflow that encompasses database searching, statistical validation, and advanced differential proteomics tools.^16,17^ To bridge the aforementioned gaps, we transformed PatternLab’s spectral-browsing module into a fully featured and interactive viewer, Spectral Cruncher, which consolidates visualization, tag generation, and AI-assisted ion prediction into a single desktop environment; this aspect not previously addressed by visualization tools. Spectral Cruncher enables de novo sequence tag extraction, automated spectral annotation, systematic tag matching and scoring, and ion-intensity prediction via SpecFormer, a transformer implementation optimized for use integrated within a graphical interface. While simmilar technologies are existing and established, our implementation unifies these within PatternLab for proteomics and thus provides a distinct focus on interactivity and accessibility. Trained on varied data sets, SpecFormer currently supports instrument-specific variants for Orbitrap and Astral analyzers with more underway, as well as a specialized version for single-cell proteomics that accounts for low ion abundance and sparse ion statistics. An integrated model manager automatically updates validated models, ensuring accessibility without manual reconfiguration.

## Methods

We introduce a series of functionalities that we refer to as the Spectral Cruncher module. Spectral Cruncher transforms PatternLab’s traditional spectral browsing into a comprehensive interactive analysis platform, offering five integrated computational tools that bridge manual spectral examination with AI-enhanced peptide identification. At its core, the Spectrum View provides visualization of raw mass spectra with dynamic annotation capabilities, allowing researchers to explore peak patterns, zoom into regions of interest, and overlay multiple ion series annotations simultaneously. The peptide spectrum matching (PSM) tool enables direct validation of peptide sequences against experimental spectra, generating theoretical fragmentation patterns for b/y ions (including neutral losses and doubly charged variants) and overlaying it on the spectrum; such functionality is already present in most tools and previous versions of PatternLab. In what follows, the Infer Tags functionality employs our graph-based sequence tagging algorithm to extract high-confidence amino acid sequences directly from spectral data, with configurable parameters for minimum tag length and mass tolerance, while automatically classifying tags as *b*-series, *y*-series, or ambiguous based on logistic regression of seven spectral features. This allows one to confront a result coming from the search engine with an unbiased possibility. The Find Tags tool complements this by enabling targeted searches for specific sequence within spectra, supporting both exact sequences and ambiguous patterns; differently than overlaying a PSM on the mass spectrum, it will search the entire spectrum for that pattern. Finally, we present the Ion Intensity Predictor which integrates our SpecFormer transformer model to predict theoretical peak intensities for any peptide sequence, providing visual comparison between predicted and observed spectra. All tools operate synchronously on the current spectrum, with results immediately reflected in the main visualization panel through color-coded peak annotations and interactive sequence tags, creating a unified environment where manual expertise and computational predictions reinforce each other rather than operating in isolation. We will now further detail our sequence tag and SpecFormer.

### Parameter Configuration Tab

The Spectral Cruncher interface provides configurable parameters to accommodate various experimental conditions and proteomics workflows Mass tolerance settings allow users to specify parts-per-million (ppm) precision for peak matching, with typical values ranging from 5 to 20 ppm depending on instrument type. The building blocks configuration enables custom amino acid definitions, including nonstandard residues and post-translational modifications. When multiple amino acids or combinations fall within the specified mass tolerance, the software automatically concatenates them into unified entries, enabling the sequence tagging algorithm to explore all viable amino acid assignments while tracing paths between spectral peaks. This automatic mass degeneracy resolution becomes particularly common when considering dipeptide options or higher ppm tolerances, ensuring comprehensive coverage of potential sequence interpretations while maintaining computational efficiency. Additional options include dipeptide gap inclusion for handling missing intermediate cleavage products, and tryptic miss cleavage allowances for incomplete enzymatic digestion. These parameters are applied consistently across all analysis modules, ensuring coherent results between manual examination and computational predictions.

### Automated Sequence Tagging and Tag Search

The de novo sequence tagging algorithm constructs a directed graph structure where nodes represent spectral peaks and edges connect peaks whose *m*/*z* differences match amino acid masses, with no circular paths allowed. For each target peak, the algorithm searches for source peaks whose *m*/*z* values differ by amino acid masses within a specified tolerance (typically 10–20 ppm). The graph construction incorporates both single amino acid transitions and, optionally, dipeptide gaps to handle missing cleavage sites or low-abundance intermediate ions. Each edge is weighted by the logarithm of the source peak intensity, with additional bonuses applied for complementary ions (peaks whose *m*/*z* values sum to the neutral precursor mass) and neutral loss patterns from residues prone to water or ammonia elimination (Ser, Thr, Asp, Glu for H_2_O; Arg, Lys, Asn, Gln for NH_3_). The algorithm employs a beam search strategy with configurable beam width to explore the most promising paths through the graph, pruning low-scoring trajectories while maintaining computational efficiency for real-time interactive use.

Tag scoring integrates multiple orthogonal features to distinguish high-confidence sequence assignments from spurious matches. The primary scoring components include a coverage score reflecting the number of high-intensity peaks incorporated into the tag, a normalized path score based on cumulative peak intensities along the sequence, and series-specific confidence metrics that classify tags as *b*-ion, *y*-ion, or undetermined. Ion series classification (i.e., determining if the tag is a *b*- or *y*-series) employs logistic regression with several features that capture distinct fragmentation behaviors. The first two features, combined, are for modeling flanking masses: (1) N-terminal flank mass—the mass between the spectrum’s start and the first tag peak, and (2) C-terminal flank mass asymmetry—calculated as C-terminal mass divided by total flank mass, then transformed via 1.0–2.0 × |0.25-ratio| to distinguish *b*-series (values near 0) from *y*-series (values near 1), as *y*-ions typically exhibit smaller C-terminal flanks due to their origin from the peptide’s C-terminus. This approach leverages empirical fragmentation patterns in MS/MS spectra, where *y*-series tags often cluster in higher *m*/*z* regions (leading to larger N-flanks and smaller C-flanks), enhancing classification accuracy by quantifying positional biases that align with known ion behaviors. The third feature (3) captures relative *m*/*z* positioning by computing where the tag’s average *m*/*z* falls within the spectrum range (normalized 0–1), exploiting the tendency for b-ions to dominate lower *m*/*z* regions and *y*-ions higher regions. Feature (4) examines 2+ fragment ion orientation; when doubly charged complementary ions are detected, their average *m*/*z* position relative to half the precursor mass indicates series type (>0.5 suggests *y*-series). Features (5) and (6) perform binary detection of diagnostic terminal ions: y1 ions (amino acid + H_2_O + H^+^) at tag start strongly indicate *y*-series, while b1 ions (amino acid + H^+^) at tag start suggest *b*-series, although b1 ions are rarely observed due to their structural instability. This rarity is indirectly accounted for and compensated in the logistic function through empirical training, where the learned coefficients adjust the feature’s impact based on its observed frequency in real spectra. Finally, feature (7) calculates the intensity-weighted complementary ratio; i.e., the fraction of total peak intensity arising from complementary ions; as higher complementary peak abundance often correlates with confident series assignment. These inputs feed into a logistic model with an intercept and learned coefficients (β_0_ = −0.40 [intercept], β_1_ = 1.10 [flank asymmetry], β_2_ = 0.55 [*m*/*z* positioning], β_3_ = 0.30 [2+ orientation], β_4_ = 1.25 [y1 detection], β_5_ = −1.10 [b1 detection], β_6_ = 0.70 [complementary ratio]), producing confidence scores where values > 0.65 indicate *y*-series.

### Ion Intensity Prediction with SpecFormer

SpecFormer is a transformer-based neural network architecture specifically designed for predicting fragment ion intensities in tandem mass spectra. The SpecFormer architecture consists of four main components: token embedding layers, positional encoding, a multihead transformer encoder, and ion-specific output projections. Peptide sequences are tokenized using a vocabulary that encompasses the 20 standard amino acids plus common post-translational modifications, specific to each model, with each residue mapped to a learnable embedding vector of dimension 128. To capture both sequence context and precursor charge state effects, the model incorporates dual embedding streams: amino acid embeddings are combined with charge state embeddings through element-wise addition before being processed by subsequent layers.

Positional encoding follows the sinusoidal approach from Vaswani et al.,^18^ enabling the model to distinguish amino acid positions within peptides of varying lengths up to 45 residues. The core transformer encoder comprises four layers, each containing multihead self-attention mechanisms with 16 attention heads and feed-forward neural networks with Gaussian Error Linear Unit (GELU) activation and hidden dimensions of 256. Layer normalization and dropout (*p* = 0.1 for embeddings, *p* = 0.1 for transformer layers) provide regularization to prevent overfitting in deeper representations of the training data sets.

The final prediction layers employ residue-specific projections that map the transformer output to ion intensity predictions. For each amino acid position, a dedicated linear layer with ReLU activation generates intensity scores for *b*- and *y*-ion series at charge states 1+ and, when required by the user, 2+, resulting in up to four intensity predictions per cleavage site. This design enables SpecFormer to capture position-dependent fragmentation patterns while maintaining computational efficiency for interactive applications.

### SpecFormer Training Methodology

SpecFormer was trained on curated data sets generated using Q-Exactive+ and Orbitrap Astral mass spectrometers (Thermo Fisher Scientific) comprising high-confidence peptide-spectrum matches validated with PatternLab for proteomics. The training data encompassed mass spectra from multiple biological samples including HeLa cells, *Mus musculus* (C57BL/6) kidney, human Glioblastoma biopsies, and human brain organoids (WT83), the later prepared at the Integrated Stem Space Cell Orbital Research (ISSCOR) center. Additionally, we developed a specialized model for single-cell proteomics using data sets from cells isolated with the cellenOne platform and analyzed on the Orbitrap Astral analyzer. All models are distributed through an integrated model manager within the software GUI, enabling automatic installation and updates as new instrument-specific variants become available. The model was trained to minimize spectral-angle loss, a cosine-based metric that quantifies the similarity between predicted and experimental fragment-ion intensity profiles. This choice is common in peptide spectrum prediction tasks because it reflects the same geometry used for spectral-library scoring. To ensure numerical stability during training, the gradient values were limited to the range [−1, 1] (a procedure known as gradient clipping that avoids uncontrolled parameter updates). Optimization used the *AdamW* algorithm—an adaptive variant of stochastic gradient descent with decoupled weight decay—combined with a *ReduceLROnPlateau* scheduler that automatically decreases the learning rate (starting from 5 × 10^–4^ with decay ≈0.93) when validation loss no longer improves. Model generalization was monitored each epoch using a held-out test data set that was never used for parameter updates.

### Deployment and Integration of SpecFormer Models

SpecFormer models are distributed as self-contained bundles containing both trained weights and configuration metadata, eliminating version compatibility issues. PatternLab’s model manager automatically detects available GPU hardware and falls back to CPU inference when necessary, ensuring broad compatibility across computational environments. Model updates are delivered through an integrated repository system that validates checksums.

## Results

### Spectral Cruncher Interface and Interactive Analysis Tools

Spectral Cruncher provides an intuitive graphical interface accessible through PatternLab’s File → Load menu when opening a Thermo.Raw file, or directly through the Utils menu. The interface presents a dual-pane layout with the Total Ion Chromatogram displayed in the upper panel, allowing users to navigate through retention times and select specific scans for detailed analysis. The lower panel houses the main Mass Spectrum viewer, where individual spectra are displayed with interactive zoom and peak selection capabilities.

The core functionality is organized within the “Analysis Tools” tab, which contains five integrated modules for spectral investigation. The “Spectrum View” tab provides the primary visualization interface with real-time peak annotation and customizable display parameters. The “PSM” (Peptide Spectrum Matching) tab enables direct validation of peptide sequences against the current spectrum, automatically generating and overlaying theoretical *b*- and *y*-ion series. The “Infer Tags” module employs our graph-based algorithm to extract sequence tags from the spectrum, while the “Find Tags” tab allows targeted searches for specific amino acid sequences within the spectral data. Finally, the “Ion Intensity Predictor” tab integrates the SpecFormer model to generate theoretical intensity predictions for any peptide sequence, enabling comparison between predicted and observed fragmentation patterns.

Figure 1 demonstrates an analysis session within Spectral Cruncher. The interface displays the annotated spectrum with identified *b*-ions (red) and *y*-ions (blue), with peak labels indicating ion type, position, and charge state.

**Figure 1:**
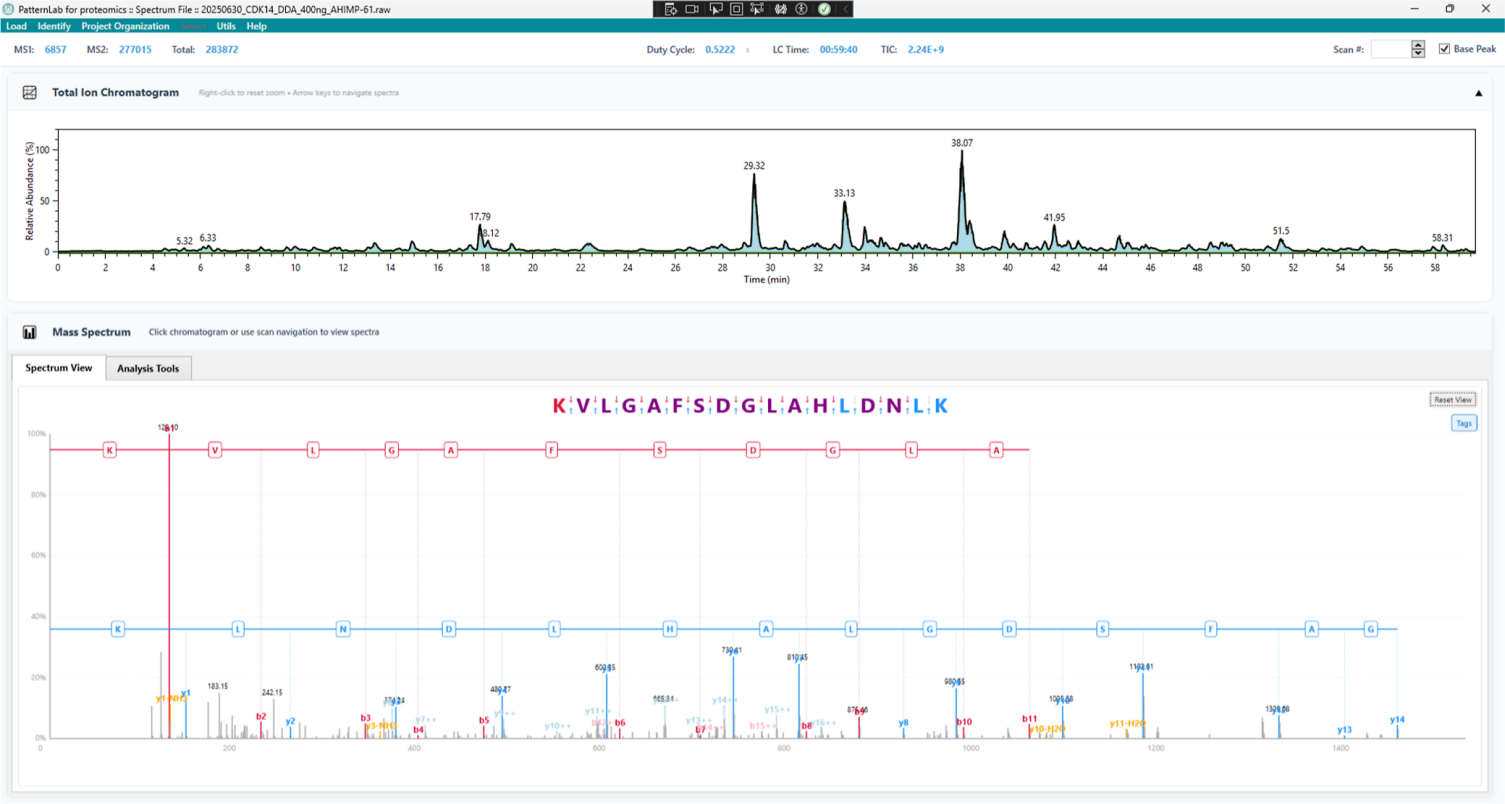
Spectral Cruncher interface showing interactive analysis of peptide KVLGAFSDGLAHLDNLK. The upper panel displays the Total Ion Chromatogram. The lower panel shows the Mass Spectrum viewer with annotated b-ions (red) and y-ions (blue) overlaid on the experimental spectrum. The peptide sequence is displayed above the spectrum with interactive fragment mapping, where each amino acid position links to its corresponding spectral peaks. The Analysis Tools tab provides access to five integrated modules for spectral investigation including spectrum viewing, PSM validation, tag inference, tag searching, and ion intensity prediction.

### SpecFormer Convergence and Effective Ion Intensity Prediction on Unseen Data Sets

SpecFormer training demonstrated excellent convergence with distinct models developed for Q-Exactive + bulk, Astral bulk, and Astral single-cell applications. During neural network training, the software iteratively learns from training data while its performance is simultaneously evaluated on a separate, independent test data set that the model never sees during learning; this test evaluation serves solely to monitor generalization capacity and provide an independent assessment in regards to overfitting. Convergence refers to the improvement and stabilization of prediction accuracy as training progresses, indicating the model has learned the underlying patterns rather than memorizing specific examples. SpecFormer’s performance is evaluated using spectral angle, which measures how similar predicted and experimental intensity patterns are, ranging from 0 radians (perfect match) to π/2 radians (∼1.57, no correlation). For intuitive interpretation, we also report cosine similarity—the cosine of the spectral angle—which ranges from 0 to 1, where values above, 0.85 are considered excellent for mass spectrometry applications.

Here, our reported angles represent the average performance across all spectra in the independent test set. The Q-Exactive + bulk model exhibited rapid and stable convergence during training with spectral angles decreasing from initial values of ∼0.63 radians to the final spectral angle of ∼0.21 radians (on the independent test set) corresponding to a cosine similarity of approximately 0.98, representing near-perfect matches between computational predictions and experimental measurements; this is superior to those reported in major manuscripts of similar tools^5^ (Figure 2). The close tracking between training and test performance throughout the learning process demonstrates robust generalization without overfitting. The Astral bulk model reached a final spectral angle of 0.411 radians (training) and 0.413 radians (test) after 144 epochs of training on the comprehensive Astral bulk data set encompassing HeLa cells, *M. musculus* tissues, and human samples. The tight convergence between training and test performance (difference of only 0.002 radians) confirms the model’s generalization capability, while the final spectral angle corresponds to a cosine similarity of approximately 0.91. The specialized Astral single-cell model converged after 218 epochs, achieving a final test spectral angle of 0.51 radians (cosine similarity ∼0.87), which, while lower than bulk proteomics models, represents strong performance given the unique challenges of single-cell data including ultralow ion abundances, sparse fragmentation patterns, and the absence of cysteine carbamidomethylation. The model demonstrated stable learning dynamics with gradual improvement and appropriate regularization, as evidenced by the controlled gap between training (0.36 radians) and test performance. All models are available for download through the graphical user interface of our software.

**Figure 2:**
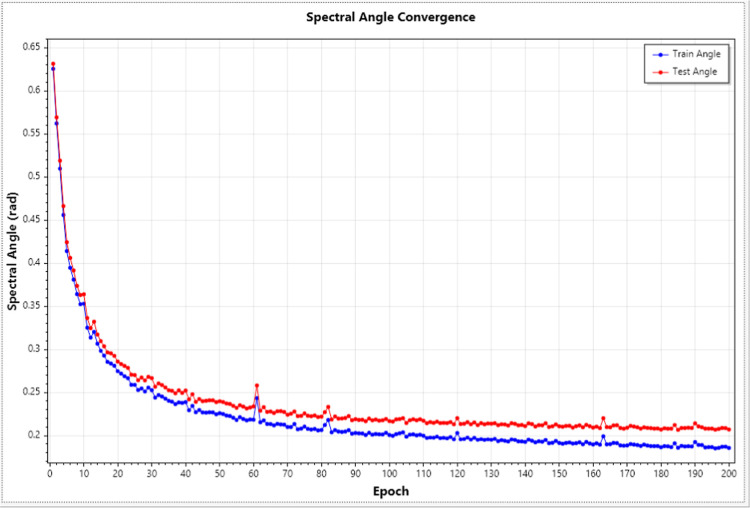
Training convergence of the SpecFormer transformer model for MS/MS ion intensity prediction. Spectral angle (radians) between predicted and observed fragment ion intensities plotted across 200 training epochs. The model demonstrates rapid convergence during the first 50 epochs, with both training (blue) and test (red) angles decreasing from ∼0.63 to ∼0.25 radians. Convergence stabilizes after epoch 100, achieving final spectral angles of 0.187 (training) and 0.207 (test). The close alignment between training and test curves throughout the entire training process indicates excellent generalization without overfitting. The final spectral angle of ∼0.21 radians corresponds to a cosine similarity of approximately 0.98, demonstrating high-quality intensity predictions. Training was performed on curated data sets from a Q-Exactive + mass spectrometer using cosine similarity loss, AdamW optimization (initial learning rate 5 × 10^–4^), and early stopping based on test set performance.

The development of instrument-specific models enables SpecFormer to capture the unique fragmentation characteristics and sensitivity profiles of different mass spectrometry platforms, ensuring optimal prediction accuracy for different experimental workflows. All models are distributed through PatternLab’s integrated model manager, providing seamless access to appropriate predictions based on the user’s instrumentation setup.

### SpecFormer Enables Visual Assessment of Ion Predictions

By entering a sequence into the SpecFormer GUI, users can generate visual interpretations of theoretical mass spectra directly within the Spectral Cruncher interface. Figure 3 exemplifies an experimental mass spectrum for the peptide LQQEATESHATESER acquired on a Q-Exactive Plus instrument, alongside its predicted counterparts generated by SpecFormer and MS^2^PIP. This side-by-side visualization allows researchers to assess the concordance between experimental and predicted fragmentation patterns, facilitating manual validation of peptide identifications.

**Figure 3:**
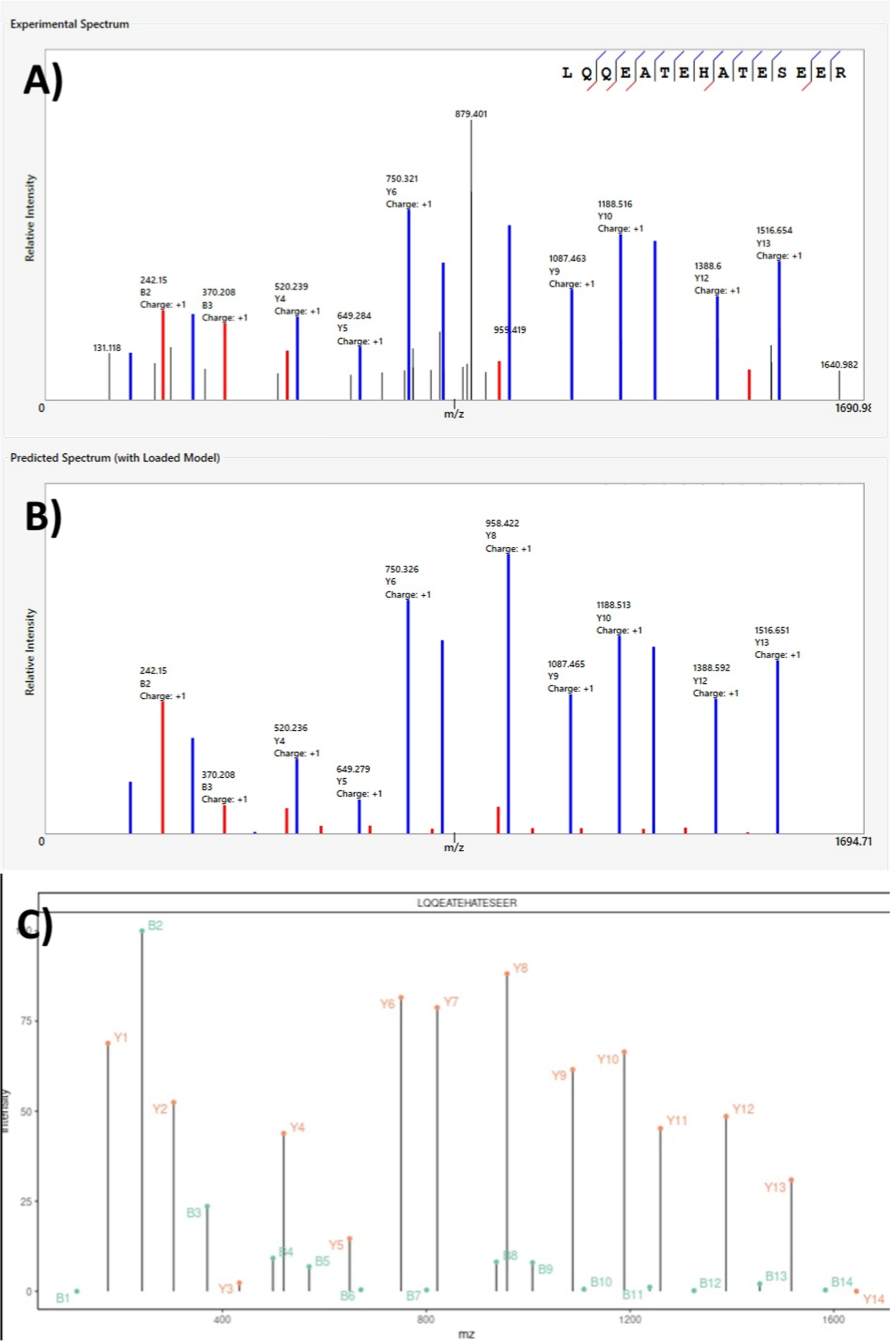
Comparison of experimental and predicted MS/MS spectra for peptide LQQEATESHATESER. MS/MS spectra obtained on a Q-Exactive Plus mass spectrometer showing (A) experimental spectrum with annotated *b*- and *y*-ion series (blue and red peaks, respectively), (B) SpecFormer-predicted spectrum with theoretical ion intensities, and (C) MS^2^PIP-predicted spectrum for comparison. All spectra show fragmentation of the doubly charged precursor ion. Peak annotations indicate ion type, position, and charge state. SpecFormer predictions demonstrate closer agreement with experimental peak intensities and relative ion abundance patterns compared to MS^2^PIP, particularly for high-intensity *y*-ions (y8, y9, y10) and the dominant b2 ion.

### Availability through PatternLab 5.1

PatternLab for proteomics was one of the first complete desktop solutions for analyzing shotgun proteomic data, providing a one-stop shop from identification to differential proteomics that has been consistently updated through the years.^13–15^ Spectral Cruncher is integrated into the development version of PatternLab for proteomics, currently designated as PatternLab 5.1; it is accessible through the File → Load menu when loading a Thermo.Raw file or in the Utils menu. As a development version, it receives frequent updates and new features, often on a weekly basis; version 5.1, freely available for academic use, is available at http://patternlabforproteomics.org/51.

## Discussion

In all, the challenge today is not whether to embrace computational power, that ship has already set sail and there’s no turning back, but rather how to equip researchers with tools that enable critical interrogation of algorithmic decisions while building the intuition to recognize when algorithms miss the mark. The path forward requires tools that transform black-box algorithms into glass boxes, where computational power amplifies rather than replaces human expertise, and where every researcher can develop the spectral intuition to know when to trust the machine and when to trust their eyes. By embedding the capabilities cited above into PatternLab, Spectral Cruncher not only allows scientists to scrutinize spectral identifications but also fosters a “computational playground” for hypothesis-driven exploration. Ultimately, this integration advances proteomics research in both routine and edge-case applications, while promoting user empowerment.

The motivation for creating SpectralCruncher emerged from a challenging case in our laboratory involving a low-abundance protein that our collaborators insisted was biologically crucial. While PatternLab’s peptide spectrum matching stringent filtering^19^ had identified a confident peptide, we needed additional supporting evidence to confidently report this protein. Our hypothesis was straightforward: given the protein’s low abundance, there should be more spectra present, however, just not of sufficient quality to pass automatic filtering thresholds. This led us to examine the vast pool of spectra that matched the theoretical precursor masses of this protein’s peptides but had been rejected by PatternLab’s statistical validation. Without proper tools for systematic interrogation, we found ourselves transported back to proteomics’ early days, by copying and manually clicking through hundreds of spectra, copying and pasting ions in web forms to obtain overlapping *b*- and *y*-ion series, searching for partial sequences that might confirm our target. This experience exposed a critical gap in modern proteomics workflows: the lack of tools to efficiently explore the twilight zone between high-confidence identifications and noise. By embedding tag generation, easy peptide spectrum matching overlays and transformer ion intensity predictions within an interactive environment that preserves manual interrogation capabilities, SpectralCruncher enables systematic exploration of these borderline spectra. The tagging algorithm, especially when combined with SpecFormer’s intensity predictions, can help validate sequence evidence in spectra.

## Conclusion

To our knowledge, we are also providing the first dedicated models for both Astral bulk and Astral single-cell proteomics—a critical advancement as single-cell analysis defies current instrumentation with ultralow ion abundances and unique challenges such as no carbamidomethylation of cysteines and sparse fragmentation patterns that confound traditional scoring algorithms. This synergy between algorithmic assistance and human judgment proves essential as proteomics ventures into increasingly challenging territories of single-cell analysis and trace protein detection. SpectralCruncher thus addresses not just a technical gap but a fundamental need: ensuring that as our field advances toward ever-deeper proteome coverage, we maintain the ability to scrutinize from the margins of our data. In bridging manual spectral interpretation with cutting-edge AI, we transform what was once an improvisation into systematic investigation, preserving the investigative spirit that once defined our field.

## Data Availability

Bulk proteomic data from human brain organoids (lineages WT83 and QX) used in this study were obtained from PRIDE data set PXD069807. Bulk proteomics from *Mus musculus* (C57BL/6) kidney and HeLa cells, as well as single-cell proteomics generated using the cellenOne platform from WT83 human brain organoids, were deposited under PRIDE accession PXD069898. Q-Exactive Plus data sets comprising human Astrocitoma biopsies (1) are available under PXD033782.
